# Alterations in plasma proteome during acute COVID-19 and recovery

**DOI:** 10.1186/s10020-024-00898-5

**Published:** 2024-08-25

**Authors:** Maciej Suski, Agnieszka Olszanecka, Aneta Stachowicz, Anna Kiepura, Michał Terlecki, Józef Madej, Marek Rajzer, Rafał Olszanecki

**Affiliations:** 1 Department of Pharmacology, Faculty of Medicine, Jagiellonian University Medical College, 16 Grzegorzecka str, Kraków, 31 531 Poland; 2 Department of Cardiology, Interventional Electrocardiology and Arterial Hypertension, Jagiellonian University Medical College, 2 Jakubowskiego str, Kraków, 30-688 Poland; 3 University Hospital in Kraków, 2 Jakubowskiego str, Kraków, 30-688 Poland

**Keywords:** COVID-19, Cardiovascular system, Plasma, Proteomics, PQ500

## Abstract

**Background:**

The severe course of COVID-19 causes cardiovascular injuries, although the mechanisms involved are still not fully recognized, linked, and understood. Their characterization is of great importance with the establishment of the conception of post-acute sequelae of COVID-19, referred to as long COVID, where blood clotting and endothelial abnormalities are believed to be the key pathomechanisms driving circulatory system impairment.

**Methods:**

The presented study investigates temporal changes in plasma proteins in COVID-19 patients during hospitalization due to SARS-CoV-2 infection and six months after recovery by targeted SureQuant acquisition using PQ500 panel.

**Results:**

In total, we identified 167 proteins that were differentially regulated between follow-up and hospitalization, which functionally aggregated into immune system activation, complement and coagulation cascades, interleukins signalling, platelet activation, and extracellular matrix organization. Furthermore, we found that temporal quantitative changes in acute phase proteins correlate with selected clinical characteristics of COVID-19 patients.

**Conclusions:**

In-depth targeted proteome investigation evidenced substantial changes in plasma protein composition of patients during and recovering from COVID-19, evidencing a wide range of functional pathways induced by SARS-CoV-2 infection. In addition, we show that a subset of acute phase proteins, clotting cascade regulators and lipoproteins could have clinical value as potential predictors of long-term cardiovascular events in COVID-19 convalescents.

**Supplementary Information:**

The online version contains supplementary material available at 10.1186/s10020-024-00898-5.

## Introduction

The impact of COVID-19 on cardiovascular disease was profound and it quickly became obvious that the severe course of COVID-19 caused cardiovascular injuries, although the involved mechanisms are still not fully recognized, linked, and understood (Boulos et al. 2023). After initial infection, the SARS-CoV-2 virus replicates in pulmonary epithelial cells and then begins to circulate, which allows it to access more distant cells that express angiotensin-converting enzyme 2 (ACE2) and other necessary cell-entry proteins (Chung et al. 2021). This is of particular importance for the cardiovascular system, since in cardiac myocytes, fibroblasts and pericytes the expression of ACE2 is constitutive or even increased in the setting of ventricular remodelling response to acute and chronic cardiovascular conditions (Gheblawi et al. 2020). This positions the heart and vasculature as an important direct and indirect target for SARS-CoV-2 and systemic COVID-19 complications, respectively. In fact, myocardial injury (MI) was evidenced through elevations in troponin levels in patients with COVID-19 and was further supported by the finding that patients with MI and COVID-19 had higher mortality and higher markers of myocardial injury (Terlecki et al. 2022). Therefore, great efforts have been made to investigate how dysfunction at the level of endothelial cells or cardiac myocytes causes injury at the level of the cardiovascular system as a whole, paying particular attention to venous and arterial thrombosis, arrhythmias, acute coronary syndromes, myocarditis, and systolic dysfunction (Jaffe et al. 2020; Chang et al. 2021; Siripanthong et al. 2022). The latter became even more necessary with the establishment of the concept of post-acute sequelae of COVID-19, referred to as long COVID (Davis et al. 2023), where blood clotting and endothelial abnormalities are believed to be the key pathomechanisms driving the impairment of the circulatory system (Haffke et al. 2022; Katsoularis et al. 2022).

The molecular pathways that govern both physiological and pathological processes that take place throughout the body result in protein secretion or leakage into the circulation. These can be traced and deciphered in a systemic fashion through in-depth characterization of a perturbed plasma proteome composition, which can be achieved by modern proteomic technologies. Plasma proteomics is of particular interest because it explores a tissue compartment that is at the crossroads of mutually percolating systemic signalling pathways. Furthermore, time-resolved changes in plasma proteome composition hold the promise of unveiling malfunctioning pathways and highlighting those that are not restored during recovery from the pathological stimulus (such as SARS-CoV-2 infection), possibly contributing to long COVID systemic complications. In fact, there are several studies using proteomic methodology for the investigation of plasma proteins to find early markers of severity and outcome of COVID-19 (Koupenova et al. 2022; Wang et al. 2022; Pagani et al. 2023; Sahin et al. 2023), discriminating SARS-CoV-2 from other infections (Palma Medina et al. 2023) and unravelling the mechanisms that comprise the onset of long COVID (Li et al. 2022; Gu et al. 2023; Hanson et al. 2023; Iosef et al. 2023; Medori et al. 2023; Peppercorn et al. 2023; Talla et al. 2023).

The aim of the presented study was to comprehensively analyse the quantitative temporal trajectories of plasma proteins of COVID-19 convalescents by means of targeted SureQuant acquisition using the PQ500 plasma protein panel. We specifically focused on cardiac parameters of the enrolled study cohort of 132 COVID-19 patients to corelate plasma protein concentration changes with their clinical characteristics.

## Materials and methods

### Patients inclusion, hospital examinations and sample collection

**Fig. 1:**
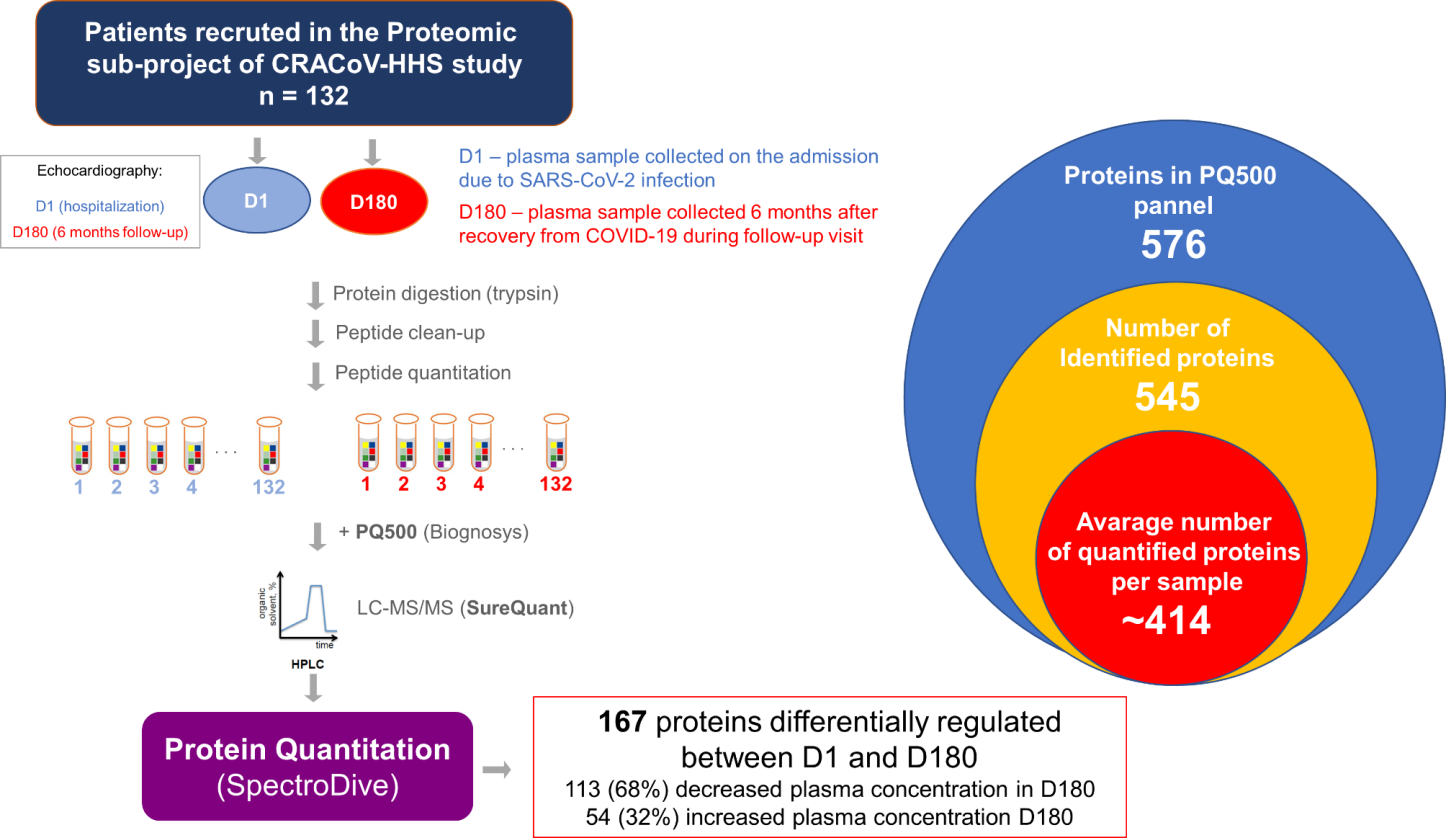
**Overview of the study design.** Quantitative plasma proteins analysis was performed on a series of samples collected during hospitalization due to SARS-CoV-2 infection and six months later during the follow-up visit. Analysis protocol employed PQ500 protein panel (Biognosys) and SureQuant acquisition

The presented study was one of the sub-projects of the CRACoV-HHS (CRAcow in CoVid pandemic — Home, Hospital and Staff) study conducted at the University Hospital in Cracow (Bioethics Committee of the Jagiellonian University approval No. 1072.6120.333.2020 dated December 7, 2020). The organizational structure, study design and setting of the CRACoV-HHS project are described in detail in (Sydor et al. 2021). Consecutive patients were recruited between the 8th of January 2021 and 30th of April 2021. The primary inclusion criteria of the CRACoV-HHS project were informed consent to participate in the study and confirmed COVID-19 infection (positive RT-PCR or antigen test), age ≥ 18 and < 75 years, while the primary exclusion criterium was the inability to give informed consent by the patient. Specific inclusion criteria for the proteomic subproject were patients aged 45 to 70 years hospitalized for COVID-19 with no previously diagnosed left ventricular systolic dysfunction with EF < 40%, a prior diagnosis of severe valvular heart disease, a history of an atherosclerotic cardiovascular event within 6 months prior to inclusion in the study (i.e. stroke, myocardial infarction, angioplasty of coronary or peripheral arteries, coronary artery bypass grafting), chronic kidney disease with eGFR < 30 mL/min/1.73 m2 at admission, active cancer and chronic inflammatory disease. We intended to include patients whose potential changes in left ventricular systolic function during follow-up could be attributed to the influence of COVID-19, not to recovery or deterioration due to known cardiac disease. The 6-month interval was chosen because studies have shown that the appropriate time for ejection fraction (EF) reassessment after revascularization is 3–6 months. Similarly, endothelial healing after denudation and endothelium regeneration takes several weeks to months. Altogether *n* = 132 patients were included in the study (patient characteristics are summarized in Table 1), who underwent echocardiography examination on the admission to the hospital and six months after discharge. A comprehensive clinical evaluation of patients, as well as the echocardiography methodology, is expounded upon in a dedicated publication (Olszanecka et al. 2023). Additionally, at these time points, blood was collected into the EDTA-coated tubes, immediately centrifuged (10 min at 2000 × g using a refrigerated centrifuge) and stored at − 80 °C for proteomic analysis. The design of the study schematic is depicted in Fig. 1.

**Table 1:** Study cohort baseline characteristics on the admission due to SARS-CoV-2 infection

Parameter	Value
Age (years)	56.1 ± 12.0
Women (n, %)	45 (34%)
Men (n, %)	87 (66%)
Weight (kg)	87.9 ± 18.4
Height (cm)	171.6 ± 10.2
BMI (kg/m2)	29.7 ± 5.3
Waist circumference (cm)	102.6 ± 16.1
Systolic BP (mmHg)	130.5 ± 15.9
Diastolic BP (mmHg)	79.9 ± 10.8
Heart Rate (/min)	73.6 ± 13.3
SpO2 (%)	90.8 ± 5.8
Hypertension (n, %)	65 (48.5%)
Diabetes (n, %)	23 (17.2%)
Ischemic heart disease (n, %)	8 (6.0%)
Atrial fibrillation (n, %)	9 (6.7%)
Previous stroke (n, %)	1 (0.7%)
PAD (n, %)	3 (2.2%)
COPD (n, %)	2 (1.5%)
Asthma (n, %)	10 (7.5%)
CKD (n, %)	3 (2.2%)
Hypothyroidism (n, %)	23 (17.2%)
Past NPL (n, %)	3 (2.2%)
Liver disease (n, %)	3 (2.2%)
Depression (n, %)	5 (3.7%)
BPH (n, %)	7 (5.2%)
ACE inhibitors (n.%)	39 (29.5%)
ARB (n.%)	13 (9.8%)
Beta blockers (n, %)	44 (33.3%)
Diuretics (n.%)	26 (19.7%)
CCB (n, %)	29 (22.0%)
Alpha blockers (n.%)	13 (9.8%)
Statins (n, %)	29 (22.0%)
ASA (n, %)	11 (8.3%)
OAC/NOAC (n, %)	4 (3.0%)
Metformin (n, %)	22 (16.7%)
SGLT2i (n, %)	2 (1.5%)
Sulfonylureas (n %)	8 (6.1%)
Insulin (n, %)	2 (1.5%)
DPP-4 inhibitors (n.%)	2 (1.5%)

BMI – body mass index, SpO2 - peripheral blood oxygen saturation, PAD – peripheral artery disease, COPD – chronic obstructive pulmonary disease, CKD – chronic kidney disease, NPL – neoplastic disease, BP – benign prostate hypertrophy, ACE – angiotensin converting enzyme, ARB – angiotensin receptor blocker, CCB – calcium channel antagonist, MRA – mineralocorticoid antagonist, OAC/NOAC – oral anticoagulant/novel oral anticoagulant, SGLT2i - sodium-glucose cotransporter-2 inhibitors, DPP-4 - Dipeptidyl-peptidase-4 inhibitors.

### Sample preparation for LC-MS/MS analysis

Plasma samples (10 µl) were processed using the sample preparation kit (Biognosys, Schlieren, Switzerland) according to manufacturer’s instructions to obtain comprehensive peptide mixtures for reproducible mass spectrometry-based proteomic measurements. After digestion with modified trypsin (Promega, Madison, WI) the samples were desalted on 96-well MiniSpin C18 columns (Harvard Apparatus, Holliston, MA), after which the peptide concentration was determined by BCA method (incubation time 2 h). The samples were then dried in a vacuum concentrator (Eppendorf, Hamburg, Germany) and resuspended at a concentration of 0.88 µg/µl with 0.1% formic acid in water. Next, samples were spiked-in with PQ500 Reference Peptide Mix (Biognosys, Schlieren, Switzerland) according to the manufacturer’s instructions. Finally, 3 µl of sample, which corresponds to ~ 2 µg sample peptides were injected on-column for LC-MS/MS measurements.

### Liquid chromatography – tandem mass spectrometry

The peptides were injected into a nanoEase M/Z Peptide BEH C18 75 μm i.d. × 25 cm column (Waters, Milford, MA) via a trap column nanoEase M/Z Symmetry C18 180 μm i.d. × 2 cm column (Waters, Milford, MA). Samples were separated using a 110 min 1–50% B phase nonlinear gradient (A phase − 0.1% FA; B phase − 80% ACN and 0.1% FA) operating at a flow rate of 250 nL/min on an UltiMate 3000 HPLC system (Thermo Scientific) and applied to a Orbitrap Exploris 480 mass spectrometer (Thermo Scientific, Waltham, MA) mass spectrometer. The main working nano-electrospray ion source (Nanospray Flex, Thermo Scientific, Waltham, MA, with PepSep adapter, Bruker Daltonics GmbH & Co. KG, Bremen, Germany) parameters were as follows: ion spray voltage 2.2 kV and transfer tube temperature 275 °C. Spectra were collected in SureQuant mode using an Xcalibur template for the PQ500 kit with modified intensity thresholds to trigger a high-resolution MS2 scan for each peptide target derived from the PQ500 survey runs using SpectroDive 11 (Biognosys, Schlieren, Switzerland).

### Mass spectrometric raw data analysis and protein quantitation

SureQuant data were analysed in SpectroDive 11 software (Biognosys, Schlieren, Switzerland) using a PQ500 reference peptide panel provided by the manufacturer with default parameters. Only those precursors that passed the q-value cut‑off of 0.01 were considered as identified and quantified and included in the differential quantitative analysis and statistical testing. The precursor quantities were calculated as the sum of all selected ions that were not removed or excluded. Cross-run normalization was performed using labelled reference peptides in a way that the signal of each target peptide was divided by the signal of the labelled reference and multiplied by the average signal of that reference across the runs. The deep learning-assisted iRT regression strategy was used to calibrate retention time based on the iRT peptides included in the PQ500 reference peptide kit. Differential protein abundance statistical testing between follow-up samples (D180) and hospitalization (D1) was performed in SpectroDive using paired Student’s tests with multiple testing correction after Storey (Storey 2002).

### Pathway annotation and functional grouping

Functional grouping and pathway annotations were performed using PINE (Sundararaman et al. 2020) with ClueGO (Bindea et al. 2009) in the Cytoscape 3.8.2 software environment (Shannon et al. 2003). CORUM-3.0 (release 03.09.2018), KEGG (release 17.02.2020), REACTOME (release 17.02.2020) and WikiPathways (release 17.02.2020) pathways were used in the analysis. The enrichment results were validated by a two-sided enrichment / depletion geometric statistical test with Bonferroni step down as the p-value correction method. The minimum and maximum levels of GO were set at 1 and 4, respectively, with the cluster criteria of 5 minimum genes comprising a minimum of 2% of the GO term. The Kappa score threshold was set at 0.4.

### Statistics

Fisher’s exact test was used to assess associations of gender distribution between subgroups of the population. All quantitative data were log_2_-transformed prior to statistical testing. The equality of variances was evaluated using the Brown-Forsythe test (multiple group comparisons) and the F test (two group comparisons), while the normality of the data and their residuals was evaluated using the Shapiro–Wilk test. The Mann-Whitney test, the Wilcoxon test, the paired T-test or Kruskal-Wallis test with a two-stage linear step-up procedure of Benjamini, Krieger and Yekutieli for multiple comparisons correction, when appropriate, were used to assign the statistical significance of the data in the GraphPad Prism 10.0.3 software (GraphPad Prism, San Diego, CA). p or q < 0.05 was considered statistically significant.

## Results

**Fig. 2:**
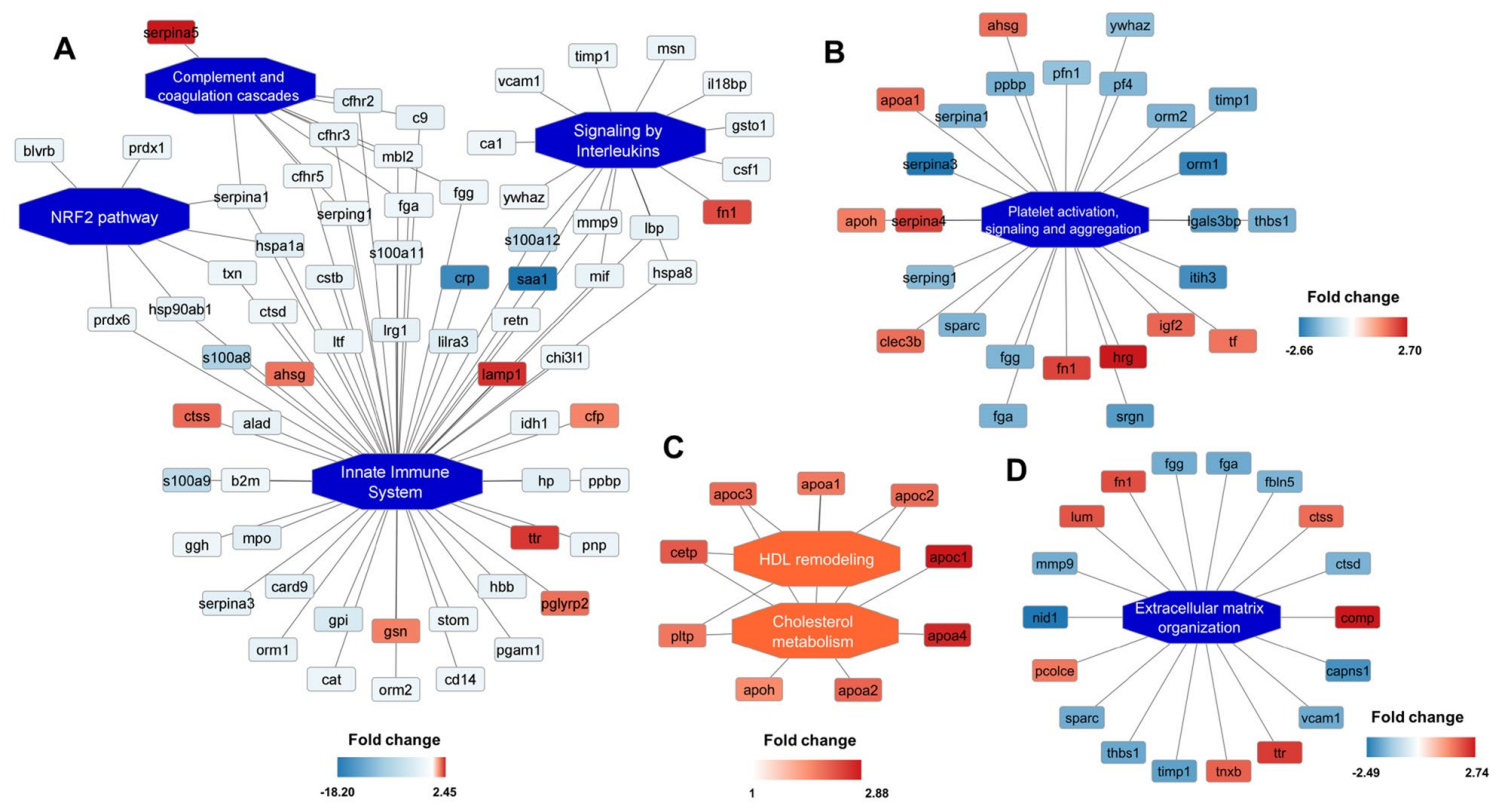
**Differential plasma proteins identification**,** quantitation and functional annotation.** The proteins included in the PQ500 panel were identified and quantified by high-resolution MS2 scans of representative proteotypic peptides (**A**), while the statistically significant regulated ones were functionally enriched to characterize affected processes (**B**)

Proteomic-targeted SureQuant analysis based on the PQ500 panel indicated that LC-MS measurements were sensitive (545 out of 576 proteins identified in the panel, 95% panel recovery) and reproducible (on average, quantification of 430 proteins in every sample, 76% of the identified proteins), allowing in-depth analysis of plasma proteome changes in COVID-19 patients (Figs. 1 and 2A). The median coefficient of variation (CV) was 44% and 40% in the D1 and D180 groups, respectively, while the significant quantitative cutoff was set for an absolute 1.5 fold change (statistical power 100%). In total, we identified 167 proteins that were differentially regulated between D1 and D180: 113 (68%) decreased, while 54 (32%) increased their plasma concentration (Fig. 1, Suppl. Tab. S1).

**Fig. 3:**
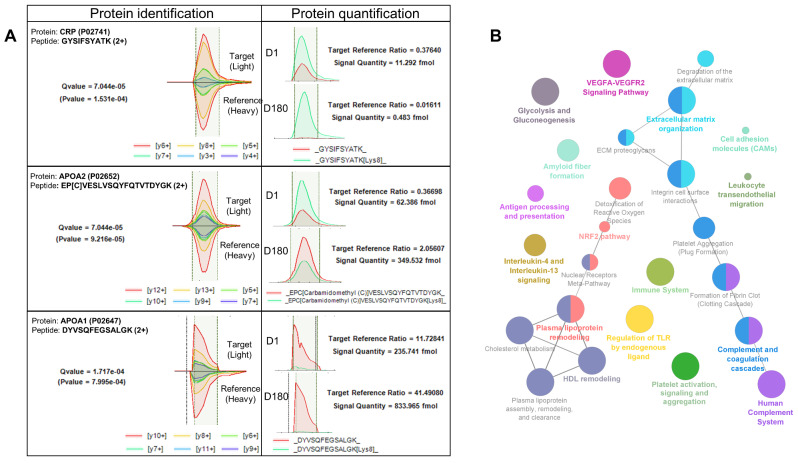
**Quantitative functional pathway enrichment.** The regulated proteins were clustered according to their biological activity to unravel the processes affected by changes in protein abundance, evidencing changes in innate immune system regulation (**A**), platelet activation (**B**), cholesterol metabolism and HDL remodelling (**C**), as well as extracellular matrix organization (**D**). The blue colour indicates repression of the protein abundance and inferred decreased activity of the corresponding pathway, while the red colour shows the opposite effect (induction)

Patients during COVID-19 exhibited substantial differences in plasma proteome composition compared to the follow-up visit after full recovery. As expected, the functional grouping and pathway annotation of the regulated proteins in plasma revealed that the pathways affected primarily during SARS-CoV-2 infection were those related to immune system activation, complement and coagulation cascades, interleukins signalling as well as platelet activation and aggregation (Figs. 2B and 3A). The proteins that were downregulated the most in COVID-19 convalescents were the acute phase proteins: C-reactive protein (CRP), serum amyloid A1 and A2 (SAA1 nad SAA2), calprotectin S100A8/A9 and other members of the S100 family (S100-A11, S100-A12) (Fig. 3A). At the same time, negative acute phase reactants such as transferrin, transthyretin, or retinol binding protein 4 were induced (Fig. 3, Suppl. Tab. S1). Furthermore, we have identified several proteins that evidence the silencing of immune cell activity in the follow-up period, including CD14 with its enhancer lipopolysaccharide binding protein (LBP), macrophage migration inhibitory factor (MIF), beta-2 microglobulin, myeloperoxidase and chemoattractant resistin. (Fig. 3A). We have also detected reduced levels of several proteins, including thioredoxin and peroxiredoxins 1 and 6, which are regulated by the activity of the nuclear factor erythroid 2-related factor 2 (NRF2) pathway (Fig. 3A). Moreover, platelet factor 4, orosomucoids 1 and 2, as well as platelet α-granule proteins: serglycin, platelet basic protein and thrombospondin 1 were all repressed in plasma six moths after COVID-19 (Fig. 3B). In addition, transferrin, which is inversely correlated with platelet function, was also upregulated in the follow-up plasma samples (Fig. 3B). Likewise, the number of proteins involved in fibrin clot formation, including fibrinogen isoforms and serine protease inhibitors: alpha-1-antitrypsin, alpha-1-antichymotrypsin, inter-alpha-trypsin inhibitor heavy chain H3, plasma protease C1 inhibitor were also downregulated in plasma during follow-up (Fig. 3A and B). On the contrary, we detected differences in plasma concentrations of several proteins, which may be interpreted as a pro-aggregatory fingerprint. For example, fibronectin (FN1) concentration was almost two times higher in follow-up plasma than during hospitalization (Fig. 3B, Suppl. Tab. S1).

Furthermore, we found that many metabolic proteins are regulated during COVID-19, with glycolytic enzymes and plasma lipoprotein regulators (especially HDL) as key features. Several protein constituents of triglycerides (apolipoproteins C1, C2 and C3), HDL (apolipoproteins A1, A2, A4 and H) as well as proteins that regulate lipid transport between triglycerides and HDL particles (phospholipid transfer protein, PLTP) and from HDL to other lipoproteins (cholesteryl ester transfer protein, CETP) were all induced during the follow-up period (Fig. 3C). These observations are in line with the literature, where a decrease in HDL and apoA1 was not only related to acute SARS-CoV-2 infection, but also the magnitude of its plasma concentration reduction can serve as a predictive marker for the severity of COVID-19 (Hilser et al. 2021; Ulloque-Badaracco et al. 2021; Chidambaram et al. 2022; Nain et al. 2022). Moreover, Gutmann et al. reported similar findings, indicating that many liver-derived proteins (including apolipoproteins) were suppressed in patients who died in the course of COVID-19 (Gutmann et al. 2021), with reduction of liver-derived miR-122 also being associated with COVID-19 mortality (Gutmann et al. 2022).

We also identified quantitative differences in the plasma concentration of several integrins, proteoglycans, and proteases involved in the organization of the extracellular matrix (Figs. 2B and 3). For example, cathepsin D (CTSD) and matrix metalloproteinase 9 (MMP9) decreased, while procollagen c-endopeptidase enhancer (PCOLCE) increased in follow up samples (Fig. 3D). Additionally, the cellular adhesion regulators: calpain small subunit 1 (CAPNS1) and fibulin 5 (FBLN5), were both down-regulated six months after COVID-19 (Fig. 3D).

**Fig. 4:**
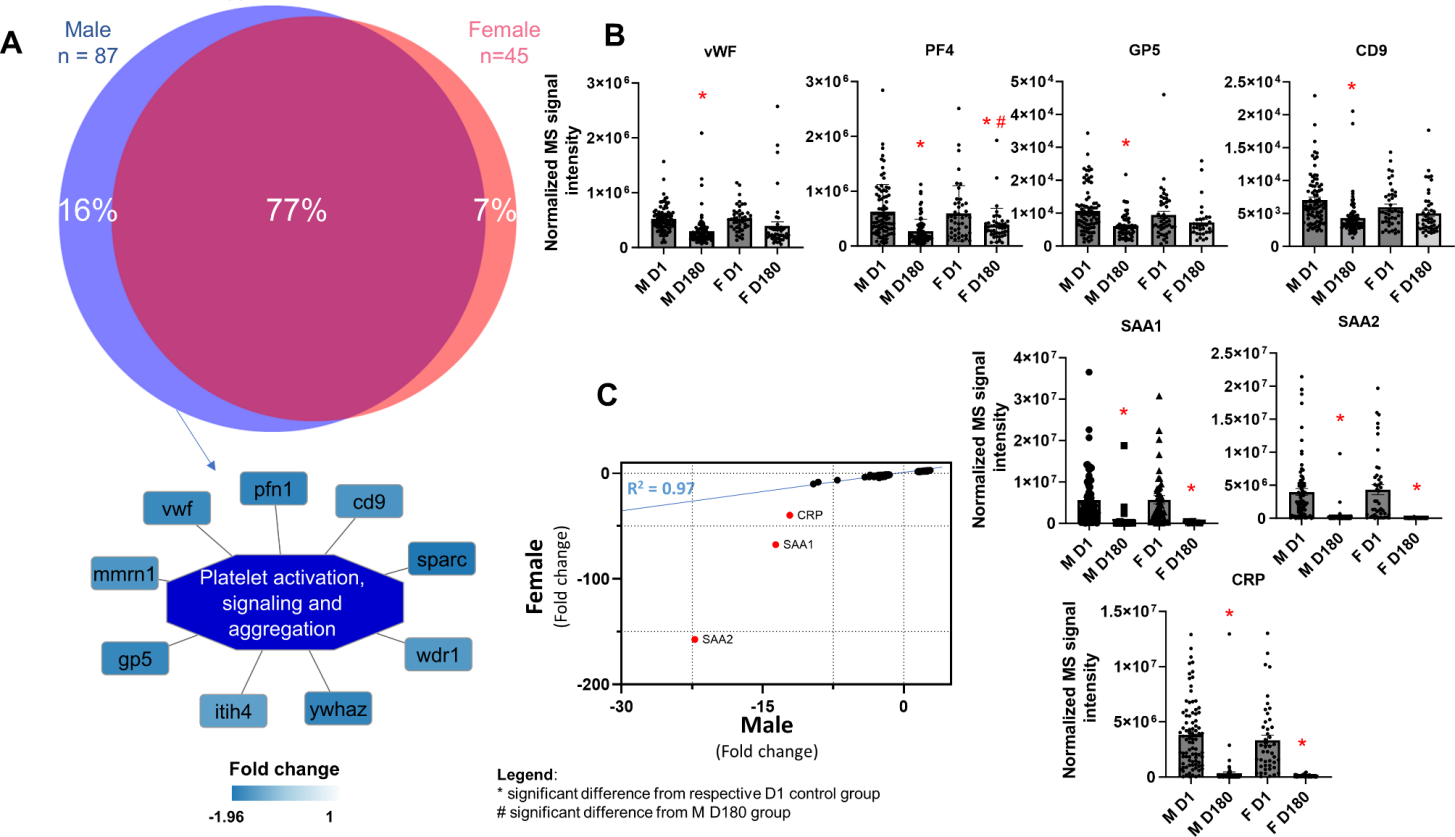
**Plasma proteins specific for gender temporal changes.** Temporal protein changes were qualitatively conserved regardless of the gender of the patients as indicated by the large overlap in the Venn diagram, however, they showed important quantitative differences. Male-specific regulated proteins functionally enriched in the platelet activation, signalling and aggregation pathways (**A**). The male subgroup was characterized with the highest platelet-related protein repression at follow-up (i.a. Von Willebrand Factor - VWF, platelet factor 4 - PF4, glycoprotein 5 - GP5, and CD9) (**B**) while the female cohort had a more evident downregulation of acute phase proteins (i.a. serum amyloid A 1 and 2 - SAA, and C-reactive protein - CRP). Furthermore, these proteins deviated from the linear correlation (R^2^ = 0.97) of the plasma proteome changes quantified in both genders during the recovery phase (**C**). M D1 (males day 1), F D1 (females day 1) – hospitalization period, M D180 (males day 180), F D180 (females day 180) – 6 month follow up

**Fig. 5:**
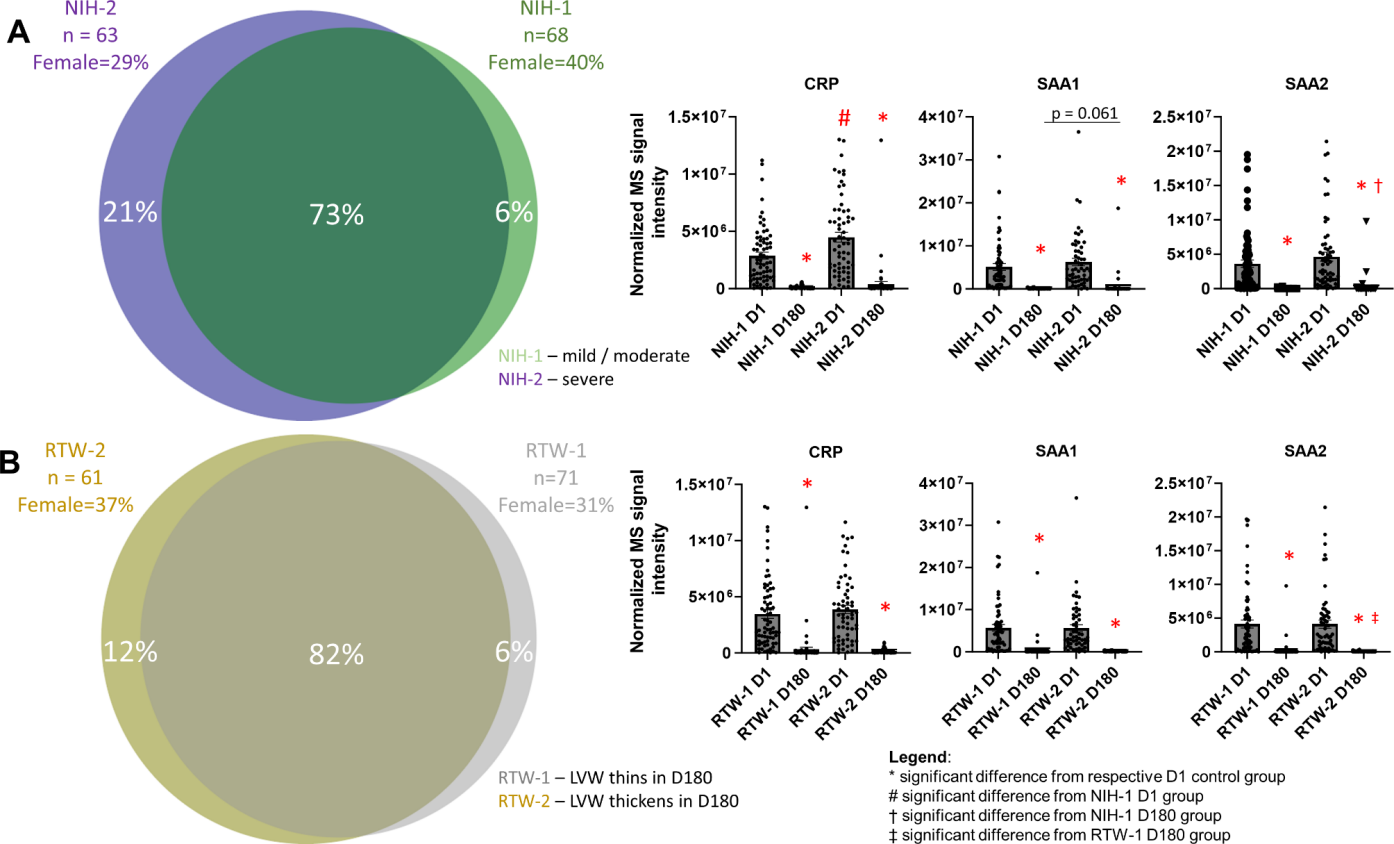
**Acute phase protein concentration differences according to clinical parameters.** The severity of the disease (NIH-1 – mild / moderate, NIH-2 – severe) (**A**) and myocardial oedema present during SARS-CoV-2 infection (**B**) caused different temporal protein changes during recovery from COVID-19. The relative wall thickness (RWT) classification indicates, that the left ventricular wall (LVW) thinned (RTW-1) or thickened (RTW-2) in the 6 month follow up period (day 180 - D180) compared to hospitalization (day 1 – D1). The large overlap of differentially regulated proteins presented by Venn diagrams evidences that plasma proteome changes in relation to the severity of the disease (NIH-1/NIH-2) or myocardial oedema (RTW-1/RTW-2) subgroups were qualitatively similar but quantitatively different, i.e. for acute phase proteins

Interestingly, we examined the plasma proteome changes in a context of several population and clinical variables of our study cohort and found that proteins involved in platelet function regulation as well as acute phase proteins follow different temporal quantitative trajectories depending on the patient’s gender (Fig. 4), the severity of the COVID-19 (Fig. 5A) and different cardiac structure and function during the acute phase of the disease (Fig. 5B). We found that plasma proteome changes were very conserved both qualitatively and quantitatively regardless of the gender of the patients, since up to 77% of differentially regulated proteins were the same in both female and male subjects (Fig. 4A). Differences in the plasma proteome of male subjects were slightly more diverse than the corresponding changes in female protein abundance (16% versus 7%, respectively), while the former subset of regulated proteins was functionally enriched in processes related to platelet activation, signalling, and aggregation (Fig. 4A).

## Discussion

The plasma proteome represents a unique biological compartment that contains proteins that are secreted from almost all tissues; therefore, it provides insight into personal health status. The main finding of the presented study was that quantitative analysis of plasma proteome changes in COVID-19 patients during active SARS-CoV-2 infection and six months later revealed a multitude of temporal differences in protein abundance during the recovery period. We report changes in protein levels for several functional domains, and these can be collectively interpreted as a progression toward recovery from acute immune system activation, platelet activation and extracellular matrix degradation. These observations are in line with other longitudinal observations of COVID-19 convalescents that incorporate proteomics and other high-throughput methodologies in long COVID studies (Gu et al. 2023; López-Hernández et al. 2023; Medori et al. 2023; Talla et al. 2023). Comprehensive assessment of the plasma proteome that allows one to capture information about the biological processes in which these proteins are involved requires a methodology that enables the simultaneous quantification of hundreds of proteins. Our study shows that modern proteomic quantitative methods based on PQ500 protein panel and SureQuant targeted acquisition can provide a thorough and comprehensive overview of aberrant plasma protein levels related to COVID-19 and their adaptive changes over time.

As expected, the main functional domain in which the majority of differentially regulated plasma proteins form follow-up COVID-19 convalescents samples are enriched is the regulation of the innate immune system, while most of the measured protein concentrations are several times lower compared to the hospitalization period (Fig. 3A). For instance, S100 proteins (downregulated during the follow-up) are involved in pro-inflammatory, antimicrobial, oxidant-scavenging and apoptosis-inducing activities. They mediate leukocyte recruitment, promote cytokine production, regulate leukocyte adhesion and migration and act as alarmins or danger associated molecular pattern (DAMP) molecules to stimulate innate immune cells by binding to pattern recognition receptors such as Toll-like receptor 4 (TLR4) (Pruenster et al. 2016; Wang et al. 2018). S100A8/A9 have been proposed as a potential diagnostic biomarker of COVID-19, since it has the ability to distinguish between mild and severe disease states (Mellett and Khader 2022). Interestingly, Guo et al. have shown that S100A8/A9 is induced during SARS-CoV-2 infection, where it mediates the activation of aberrant neutrophils that cause uncontrolled pathological damage and onset of COVID-19, while inhibition of S100A8/A9 could reduce the stimulation of these cells and restore their antiviral responses (Guo et al. 2021). Furthermore, Colicchia et al. have recently identified GPIbα as the receptor for S100A8/A9 in platelets that induces their procoagulant phenotype (Colicchia et al. 2022), evidencing that plasma S100A8/A9 is one of the link points between the immune system and platelet function. It is important to note that our results are in line with the recent studies linking plasma inflammatory protein alterations (Hanson et al. 2023), as well as changes in the peripheral blood mononuclear cell proteins (Peppercorn et al. 2023) long COVID neurologic manifestations elicited by the immune system.

The NRF2 pathway has been widely implicated in COVID-19, considering that one of the causes of long COVID is believed to be related to altered expression of antioxidant enzymes and cytoprotective proteins regulated by NRF2, which in turn results in oxidative stress (Muchtaridi et al. 2022). In our study, we have identified dynamic changes in various proteins that are under the regulation of the NRF2 pathway, including the reduction of thioredoxin and peroxiredoxins 1 and 6 at follow-up. Activation of the NRF2 pathway is also being postulated as one of the potential therapeutic strategies against the cytokine storm induced in COVID-19 (Cuadrado et al. 2020; Singh et al. 2021).

Intriguingly, we found that the concentration of fibronectin increased during the follow-up period. As a result of activation of the blood coagulation cascade by tissue factor plasma fibronectin crosslinks with fibrin fibers in a reaction catalyzed by coagulation factor XIII (FXIIIa) (Matsuka et al. 1997). More, fibronectin can be assembled into the fibrillar extracellular matrix (fibrillogenesis), which stabilizes platelet aggregates and enhances their cohesion (Olorundare et al. 2001). Intravascular microscopy has shown that plasma fibronectin can replace fibrinogen to occlude the damaged vessel and play an important role in the formation of platelet thrombus (Ni et al. 2000). These thrombi have the characteristics of reduced platelet adhesion and frequent embolization, with reduced platelet/platelet cohesion (Ni et al. 2003). Intriguingly, fibronectin aggregates have recently been shown to modulate pro-inflammatory and anti-inflammatory features of macrophages (Sikkema et al. 2018), demonstrating that this glycoprotein possesses important functionalities that link platelet function and immune cell response. Interestingly, we observed a 2-fold increase in plasma concentration of histidine-rich glycoprotein (HRG) (Fig. 3B), a protein being a negative acute phase reactant (Saigo et al. 1990). However, HRG interacts with many ligands, including heparin, thrombospondin, fibrinogen and plasminogen and through these interactions it mediates inhibition of fibrinolysis and reduction of inhibition of coagulation, which indicate a strong prothrombotic potential (Poon et al. 2011). Furthermore, HRG is involved in modulating immune complex (IC) formation, as well as in regulating FcγR function by helping phagocytes remove IC and dying cells (Gorgani et al. 1999; Poon et al. 2010). Together, the plasma proteome changes measured six months after COVID-19 evidence a complex network of different platelet regulators and the overall interpretation of these changes remains unclear and certainly requires further studies. The latter is of great importance, since thromboinflammation, persistent platelet activation and hyperactivity, thrombotic endotheliitis and abnormalities of coagulation in COVID-19 survivors are postulated to drive the onset of long COVID (Martins-Gonçalves et al. 2022; Nicolai et al. 2023; Turner et al. 2023a, 2023b).

Finally, our targeted plasma proteomic measurements aggregate the regulated proteins into the extracellular matrix (ECM) domain, where most of the observed changes can be attributed to inhibition of ECM degradation and reorganization (Fig. 3D). Interestingly, thrombospondin-5 (cartilage oligomeric matrix protein, COMP), which is essential to maintain a contractile / differentiated phenotype of vascular smooth muscle cells (VSMC) under physiological and pathological stimuli (Wang et al. 2010), was up-regulated during recovery from COVID-19 (Fig. 3D). Furthermore, from the perspective of ECM, fibronectin induction and its increased activity can be interpreted as an important regulator of tissue repair processes where FN1 allows communication between the intracellular and extracellular environment (To and Midwood 2011). In this regard, it is worth noting that FN1 seems to operate in a relation with tenascin XB (TNXB) also induced in the follow-up plasma samples (Fig. 3D). The latter protein participates in matrix maturation during wound healing and has anti-adhesive effects, as opposed to fibronectin which is adhesive (Matsumoto and Aoki 2020).

Next, we incorporated several clinical variables from the COVID-19 convalescents cohort to assess whether a specific plasma protein fingerprint can distinguish patients with different clinical status observed during active SARS-CoV-2 infection and throughout recovery up to six months after hospitalization. We found that the platelet-related protein panel (VWF, PF4, GP5, and CD9) was reduced to a greater extent in males than in females (Fig. 4B). Our proteomic results clearly suggest that, apart from direct platelet-related mechanisms, there are also other indirect processes that regulate platelet function and indicate that they might be regulated in a gender-specific manner, however, this attractive hypothesis requires further studies. Additionally, the acute phase proteins (CRP, SAA1 and SAA2) deviated from the linear correlation (R^2^ = 0.97) of the plasma proteome changes quantified in both genders, indicating a 3- to 6-fold difference in concentrations measured six months after COVID-19 (Fig. 4C). We extracted these three proteins and evaluated their plasma concentration differences according to the patient’s clinical status. As expected, we found that their levels were similar (SAA1 and SAA2) or increased (CRP) to a greater extent in patients with more severe infection symptoms (NIH-2 group) during infection compared to those with mild to moderate characteristics (NIH-1 group). Similarly, plasma levels of these proteins measured during the follow-up visit were lower in the NIH-1 group compared to their NIH-2 counterparts (Fig. 5A). Additionally, we showed that plasma levels of CRP, SAA1, and SAA2 are different six months after COVID-19 recovery in patients in whom changes in left ventricular mass and geometry were detected in acute disease and follow-up, compared to those who did not show significant changes in cardiac structure and function (Fig. 5B). Changes in left ventricular mass and relative wall thickness (RWT) estimated by echocardiography during hospitalization and follow-up distinguished patients whose left ventricular wall thinned during the recovery phase, indicating the onset of oedema during hospitalization (RWT-1 group). For these COVID-19 convalescents, we found higher plasma concentrations of CRP, SAA1, and SAA2 six months after COVID-19. While most COVID-19 patients exhibited detectable changes in cardiac performance in echocardiography during the acute phase of the disease, reflecting adaptation to the increased hemodynamic stress associated with acute inflammatory lung disease, these changes did not progress to the development of left ventricular systolic dysfunction or heart failure during the follow-up period. However, in subjects displaying significant alterations in left ventricular geometry, which imply myocardial oedema during the acute phase of the disease, the long-term consequences are still unknown. Although acute phase proteins are typically considered nonspecific inflammatory markers with limited diagnostic utility for cardiac injury, they serve as robust independent predictors of cardiovascular risk and events (Ahmed et al. 2012). Our data suggest that monitoring time-dependent changes in plasma concentration after COVID-19 may be important for the prediction of long-term cardiovascular complications in patients with severe infection symptoms with simultaneous reversible cardiac morphology aberrations. Intriguingly, we identified that the protease C1 inhibitor was downregulated in COVID-19 convalescents (Fig. 2). Taking into account its involvement in bradykinin production and function, C1 inhibitor deficiency was linked to the pathophysiology of angioedema (Cicardi et al. 2014) and it was hypothesized that it was involved in SARS-CoV-2-elicited angioedema events (Grewal et al. 2020; Belbézier et al. 2021; Szilágyi et al. 2023; Grumach et al. 2021). Unfortunately, in our study population, the number of such events was insufficient to draw any conclusions about the possible consequences of the reduction of the C1 inhibitor plasma level during recovery.

Clearly, the major limitation of our study is that proteomic findings were not confirmed by a validation cohort, in which, by means of a similar methodology, we would be able to obtain similar results. Another important issue is that 70% of patients received heparin during hospitalization. As heparin is known regulator of endothelial cell-related factors as well as secretory activities of other vascular and blood cells we cannot rule out, nor precisely assess the impact of heparin-induced plasma proteome changes concomitant with those originating in response to SARS-CoV-2 infection. Importantly, the GLM analysis indicated that age and disease severity as covariates significantly influenced CRP, SAA1, and SAA2 levels in the context of gender-specific differences observed in our study, therefore, these observations need to be verified in further dedicated studies. Furthermore, we did not assess the SARS-CoV-2 RNAemia during hospitalization, so we cannot employ this important parameter in our proteomic results. As reported by Gutmann et al., RNAemia was associated with higher 28-day intensive care unit (ICU) mortality and was comparable in predictive performance to the best protein predictors. The machine learning approach identified RNAemia combined with age or pentraxin-3 plasma levels as the best binary signatures associated with 28-day ICU mortality. Moreover, different longitudinal protein quantitative trajectories were associated with the presence of SARS-CoV-2 RNAemia (Gutmann et al. 2021). These results strongly suggest that RNAemia is an important modulator of plasma proteome changes in COVID-19 convalescents.

In conclusion, an in-depth targeted proteome investigation demonstrated substantial changes in plasma protein composition in patients during and after COVID-19, evidencing a variety of functional pathways induced by SARS-CoV-2 infection that follow different quantitative trajectories over time. Our data point to mechanisms related to platelet activation and extracellular matrix organization, which exhibit a protein fingerprint of mutually intertwined processes that may not be fully resolved to restore cardiovascular physiology. Time-dependent traits of the acute phase proteins suggest that they may possess clinical value as potential predictors of long-term cardiovascular events in COVID-19 convalescents.

### Electronic supplementary material

Below is the link to the electronic supplementary material.

Supplementary Material 1

## Data Availability

No datasets were generated or analysed during the current study.

## Declarations

### Dara availability and materials

The datasets created and analysed during the current study are available from the corresponding author on reasonable request.

## Abbreviations

ACE2: angiotensin converting enzyme 2
apoA1: apolipoprotein A1
CAPNS1: calpain small subunit 1
CD: cluster of differentiation
CETP: cholesteryl ester transfer protein
COMP: cartilage oligomeric matrix protein
COVID-19: coronavirus disease 2019
CRP: C-reactive protein
CTSD: cathepsin D
CV: coefficient of variation
DAMP: danger associated molecular pattern
ECM: extracellular matrix
EF: ejection fraction
eGFR: estimated glomerular filtration rate
FBLN5: fibulin 5
FcγR: Fc gamma receptor
FN1: fibronectin
FXIII: acoagulation factor XIII
GO: gene ontology
GP: glycoprotein
HDL: high-density lipoprotein
HRG: histidine rich glycoprotein
IC: immune complexes
ICU: intensive care unit
LBP: lipopolysaccharide binding protein
LC-MS: liquid chromatography – mass spectrometry
LVW: left ventricular wall
MI: myocardial injury
MMP9: matrix metalloproteinase 9
MS2: mass spectrometric peptide fragmentation spectrum
NET: neutrophil extracellular trap
NRF2: nuclear factor erythroid 2-related factor 2
PCOLCE: procollagen c-endopeptidase enhancer
PF4: platelet factor 4
PLTP: phospholipid transfer protein
RT-PC: Real-time polymerase chain reaction
RWT: relative wall thickness
SAA: serum amyloid A
SARS-CoV-2: severe acute respiratory syndrome coronavirus 2
TLR4: toll-like receptor 4
TNXB: tenascin XB
VSMC: vascular smooth muscle cell
VWF: Von Willebrand Factor
